# Interventions to improve linear growth during complementary feeding period for children aged 6-24 months living in low- and middle-income countries: a systematic review and network meta-analysis

**DOI:** 10.12688/gatesopenres.13083.2

**Published:** 2020-09-24

**Authors:** Jay J. H. Park, Ofir Harari, Ellie Siden, Louis Dron, Noor-E Zannat, Joel Singer, Richard T. Lester, Kristian Thorlund, Edward J. Mills

**Affiliations:** 1 Experimental Medicine, Department of Medicine, University of British Columbia, Vancouver, BC, V6T 1Z3, Canada; 2 MTEK Sciences, Vancouver, BC, V5Z1J5, Canada; 3 School of Population and Public Health, University of British Columbia, Vancouver, BC, V6T 1Z3, Canada; 4 Data and Methodology Program, CIHR Canadian HIV Trials Network, Vancouver, BC, V6Z 1Y6, Canada; 5 Department of Health Research Methodology, Evidence, and Impact, McMaster University, Hamilton, ON, L8S 4K1, Canada

**Keywords:** Complementary feeding, low- and middle-income countries, network meta-analysis, height-for-age, stunting, child development

## Abstract

**Background:** Optimizing linear growth in children during complementary feeding period (CFP) (6-24 months) is critical for their development. Several interventions, such as micronutrient and food supplements, deworming, maternal education, and water, sanitation and hygiene (WASH), could potentially be provided to prevent stunting, but their comparative effectiveness are currently unclear. In this study, we evaluated comparative effectiveness of interventions under these domains on child linear growth outcomes of height-for-age z-score (HAZ) and stunting (HAZ <-2SD)

**Methods: **For this study, we searched for low- and middle-income country (LMIC)-based randomized clinical trials (RCTs) of aforementioned interventions provided to children during CFP. We searched for reports published until September 17, 2019 and hand-searched bibliographies of existing reviews. We performed random-effects network meta-analysis (NMA) for HAZ and stunting.

**Results: **The evidence base for our NMA was based on 79 RCTs (96 papers) involving 81,786 children. Among the micronutrients, compared to standard-of-care, iron + folic acid (IFA) (mean difference =0.08; 95% credible interval [CrI]: 0.01, 0.15) and multiple micronutrients (MMN) (mean difference =0.06; 95%CrI: 0.01, 0.11) showed improvements for HAZ; MMN also reduced the risks for stunting (RR=0.86; 95%Crl: 0.73, 0.98), whereas IFA did not (RR=0.92; 95%Crl: 0.64, 1.23). For food supplements, flour in the caloric range of 270-340 kcal (RR=0.73; 95%Crl: 0.51, 1.00) and fortified lipid-based nutrient supplements (LNS) containing 220-285 kcal (RR=0.80; 95%Crl: 0.66, 0.97) decreased the risk of stunting compared to standard-of-care, but these interventions and other food supplements did not show improvements for HAZ. Deworming, maternal education, and WASH interventions did not show improvements for HAZ nor stunting.

**Conclusion:** While we found micronutrient and food supplements to be effective for HAZ and/or stunting, the evidence base for other domains in this life stage was limited, highlighting the need for more investigation.

**Registration: **PROSPERO CRD42018110449; registered on 17 October 2018.

## Introduction

Linear growth is a marker of healthy childhood progression, closely linked with neurodevelopment in early life ^1^. Despite global improvements in maternal, newborn, and child health (MNCH), the rate of children that fail to achieve their linear growth potential in low- and middle-income countries (LMICs) is high ^2^. Prevention of linear growth faltering, also known as stunting (low height for age), during the complementary feeding period (6 to 24 months of age) is critical, since stunting during this life stage can have immediate, short- and long-term consequences that are difficult to reverse ^3– 5^. Continued malnutrition in children experiencing stunting can result in increased susceptibility and frequency to infections, as well as enhanced likelihood of cognitive, motor, and language impairment ^2, 6^. In later life, stunted children may also experience reduced life chances such as poor academic performance that may affect future earnings and increased risk for chronic diseases, including obesity if accompanied by excessive weight gain in adulthood ^5, 7– 9^.

 As children begin to wean off breastfeeding, there is a critical and continual need to ensure proper nutrition, hygiene, control of infectious diseases, and overall care during the complementary feeding period. Despite multiple factors playing a role in child’s linear growth, the majority of the reviews concerning linear growth for children during this life period in the past have focused on a single intervention domain (e.g. micronutrients) ( Table 1). The comparative effectiveness of interventions is not clear across multiple domains, such as micronutrients, food supplements, deworming, maternal education, and water, sanitation, and hygiene (WASH) that could be important solutions to optimize linear growth during this life period. Additionally, all of the existing reviews have implemented a traditional pairwise meta-analysis that only allows for comparison between two interventions that have directly been compared head-to-head in clinical trials. Given that the majority of trials have used placebo or other comparators with limited clinical interest, the utility of pairwise meta-analysis can be limiting, particularly when assessing the broad sets of interventions that could be provided during the complementary feeding period.

**Table 1.** Existing reviews on interventions for children aged 6 to 24 months.

Study ID	Title	Interventions domains	No of studies	Types of studies included
Dangour 2013 ^20`^	Interventions to improve water quality and supply, sanitation and hygiene practices, and their effects on the nutritional status of children (Review)	WASH	14	RCTs, cluster-RCTs, quasi- and non-randomised trials, controlled cohort or cross-sectional studies and historically controlled studies
Darlow 2016 ^21^	Vitamin A supplementation to prevent mortality and short- and long-term morbidity in very low birth weight infants (Review)	Micronutrient: Vitamin A	10	RCTs
Das 2013 ^22^	Micronutrient fortification of food and its impact on woman and child health: a systematic review	Micronutrients	201	RCTs, quasi-experimental and before-after studies.
De-Regil 2011 ^23^	Intermittent iron supplementation for improving nutrition and development in children under 12 years of age (Review)	Micronutrient: Iron (intermittent)	33	RCTs and quasi-RCTs with either individual or cluster randomisation
De-Regil 2013 ^24^	Home fortification of foods with multiple micronutrient powders for health and nutrition in children under two years of age (Review)	Home fortification	8	RCTs or quasi-RCTs
Devakumar 2016 ^25^	Maternal antenatal multiple micronutrient supplementation for long-term health benefits in children: a systematic review and meta-analysis.	Micronutrient: MMS	9	RCTs, cluster-RCTs
Gaffey 2013 ^26^	Dietary management of childhood diarrhea in low- and middle-income countries: a systematic review.	Diet for diarrhea management	29	RCTs
Gough 2014 ^27^	The impact of antibiotics on growth in children in low and middle income countries: systematic review and meta- analysis of randomised controlled trials	Antibiotics	10	RCTs
Imdad 2011 ^28^	Effect of preventive zinc supplementation on linear growth in children under 5 years of age in developing countries: a meta- analysis of studies for input to the lives saved tool	Micronutrient: Zinc	36	RCTs
Imdad 2017 ^29^	Vitamin A supplementation for preventing morbidity and mortality in children from six months to five years of age (Review)	Micronutrient: Vitamin A	45	RCTs, Cluster-RCTs
Kristjansson 2015 ^30^	Food supplementation for improving the physical and psychosocial health of socio- economically disadvantaged children aged three months to five years	Food supplementation	26	RCTs and studies with historical controls
Lassi 2013 ^31^	Impact of complementary food and education on complementary food on growth and morbidity of children less than 2 years of age in developing countries: a systematic review	Complementary foods	16	RCTs, nonrandomized trials
Matsungo 2017 ^32^	Lipid-based nutrient supplements and linear growth in children under 2 years: a review	Lipid supplements	7	RCTs
Mayo-Wilson 2014 ^33^	Zinc supplementation for preventing mortality, morbidity, and growth failure in children aged 6 months to 12 years of age (Review)	Micronutrient: Zinc	80	RCTs
Pasricha 2013 ^34^	Effect of daily iron supplementation on health in children aged 4-23 months: a systematic review and meta-analysis of randomised controlled trials.	Micronutrient: Iron	35	RCTs
Petry 2016 ^35^	The Effect of Low Dose Iron and Zinc Intake on Child Micronutrient Status and Development during the First 1000 Days of Life: A Systematic Review and Meta-Analysis.	Micronutrient: Iron + zinc	90	RCTs or quasi-RCTs
Salam 2013 ^36^	Effectiveness of micronutrient powders (MNP) in women and children	Micronutrient: Micronutrient powders	17	RCTs
Sguaseero 2012 ^37^	Community-based supplementary feeding for promoting the growth of children under five years of age in low and middle income countries (Review)	Community-based supplementary feeding	8	RCTs
Taylor-Robinson 2015 ^38^	Deworming drugs for soil-transmitted intestinal worms in children: effects on nutritional indicators, haemoglobin and school performance (Review)	Deworming	45	RCTs or quasi-RCTs

Network meta-analysis, as an extension of conventional pairwise meta-analysis, allows for comparisons of interventions that have not been directly compared in head-to-head randomized clinical trials within a single analysis ^10, 11^. While network meta-analysis is new to MNCH, this technique has been endorsed by the World Health Organization (WHO) to support the development of intervention guidelines in global health ^11^, with the past WHO guidelines on HIV drug and behavioral therapies and direct acting agents against hepatitis C having been formulated using network meta-analysis ^12– 14^. This study uses a systematic review and network meta-analysis to determine the comparative effectiveness across intervention domains in micronutrient supplements, food supplements, deworming, maternal education, and WASH interventions on HAZ and stunting for children aged 6–24 months living in LMICs. 

## Methods

The protocol for this study was registered on PROSPERO ( CRD42018110449). The study was conducted according to the Preferred Reporting Items for Systematic Reviews and Meta-Analysis (PRISMA) extension to network meta-analysis ^15^.

### Search strategy and selection criteria

Two-way sensitivity searches were conducted for this study. First, key MNCH articles, including Bhutta *et al.* ^16^, and the Lancet 2013 umbrella review on evidence-based interventions ^2, 17^, were reviewed for relevant systematic reviews and trials. A hand-search of the bibliography of Bhutta *et al.* ^16^ was done to identify relevant systematic reviews and trials, and searches were done on PubMed and the Cochrane Database of Systematic Reviews to identify additional reviews that were published after 2013. The list of published reviews relevant to this study is provided in Table 1. 

As the second step, a full comprehensive search of literature was conducted from database inception up to September 17, 2019. The Cochrane Central Register of Controlled Trials, Embase, and MEDLINE were searched to identify relevant trials and any additional relevant reviews that were missed in the prior step (search terms are provided in *Extended data*, Supplementary Tables 1, 2, and 3) ^18^. Hand searches were done on the reference lists from the relevant reviews identified to improve the sensitivity of this study’s search.

Table 2 summarizes the Population, Intervention, Comparator, Outcomes, and Study Design (PICOS) criteria used to guide the study selection for our systematic literature review. In brief, randomized clinical trials that assessed interventions’ comparative effectiveness on HAZ and/or stunting (a HAZ score of less than 2 SDs below the WHO Child Growth Standards median) ^19^ for children aged 6 to 24 months living in LMICs. The intervention domains of focus included: micronutrient supplements, food supplements, deworming, maternal education, and WASH interventions. Non-English-language studies were excluded. Four reviewers (JJHP, ES, LD, and NEZ) independently reviewed all abstracts and proceedings identified in the systematic search. The same reviewers independently conducted the full-text review. Any discrepancies were resolved by discussion, and if a resolution could not be achieved, a fifth reviewer (KT) settled the disagreements.

**Table 2.** Population, interventions, comparator, outcomes, and study design (PICOS) criteria.

Category	Inclusion criteria
Population	Children of age 6 to 24 months, living in low- and middle-income countries
Intervention	• Micronutrient & calcium supplementation to children • Food supplementation to children • Deworming • Maternal education • Any water, sanitation and hygiene (WASH) intervention
Comparators	• Placebo • Standard-of-care (if applicable) • No intervention • Any of the interventions listed above as monotherapy or in combination that can be used for indirect comparison
Outcomes	At least one of the following outcomes (reported after at least 3 months): • Height for age z-score (HAZ) • Proportion of stunted (HAZ < -2SD)
Study Design	Randomized clinical trials
Other	Published in the English language

Using a standardized data sheet in Microsoft Excel, four investigators (JJHP, ES, LD, and NEZ) independently extracted data for study characteristics, interventions used, participant characteristics at baseline, and outcomes from the final subset of eligible studies. Any discrepancies observed during data extraction were resolved by consensus achieved through discussion.

### Data analysis

The network meta-analyses for this study were done using the Bayesian framework in *R* via the R2WinBUGS v14 package ^39, 40^. Bayesian models were performed according to the guideline of NICE Technical Support Document 2 (TSD2) ^41^. Estimates of comparative effectiveness were measured using mean differences in HAZ with associated 95% credible intervals (95% CrI); and risk ratios (RRs) with associated 95% CrI for the stunting outcome. As heterogeneity was anticipated, random-effects network meta-analysis models were performed. In all models, an empirically informative heterogeneity prior distribution were used, as suggested by Rhodes *et al.* ^42^ for continuous outcomes and Turner *et al.* ^43^ for dichotomous outcome, to stabilize the estimation of heterogeneity in the face of low number of trials per comparison in the network. The model selection was informed by using the deviance information criterion (DIC) and the deviance-leverage plots that could help identify outlier(s) in terms of model fit, in accordance to the NICE TSD2 recommendations ^41^.

For both HAZ and stunting, the primary analysis included both cluster and non-cluster randomized clinical trials (with the unit of randomization performed at the individual level). Within the cluster trials included in our network meta-analysis, an average value of ICC of 0.0505 was reported. Thus, in order to adjust for clustering effects of the cluster trials, a conservative intracluster correlation coefficient of 0.05 was assumed and we used inflated variances accordingly for the continuous outcome, and adjusted the sample sizes and the number of cases for the dichotomous outcome, following the principles recommended by Uhlmann *et al.* ^44^ Sensitivity analyses were conducted for each outcome by using non-cluster randomized clinical trials only in the analyses. Full details of the statistical approaches are provided in the *Extended data* (Supplementary Table and figures file) ^18^.

### Risk of bias within and across studies

Each full text article was evaluated for reporting quality according to the Cochrane Risk of Bias Tool ^45^. Bias was evaluated using the Cochrane Risk of Bias tool in the areas of selection, performance, detection, attrition, reporting, and other sources of bias. Over 60% of the studies exhibited low bias in terms of attrition, selection, and reporting bias. Sources of detection and performance bias were unclear in about 25–30% of the studies. The risk of bias assessment within and across studies are provided in the *Extended data* (Supplementary Table 8) ^18^. 

## Results

**Figure 1.**
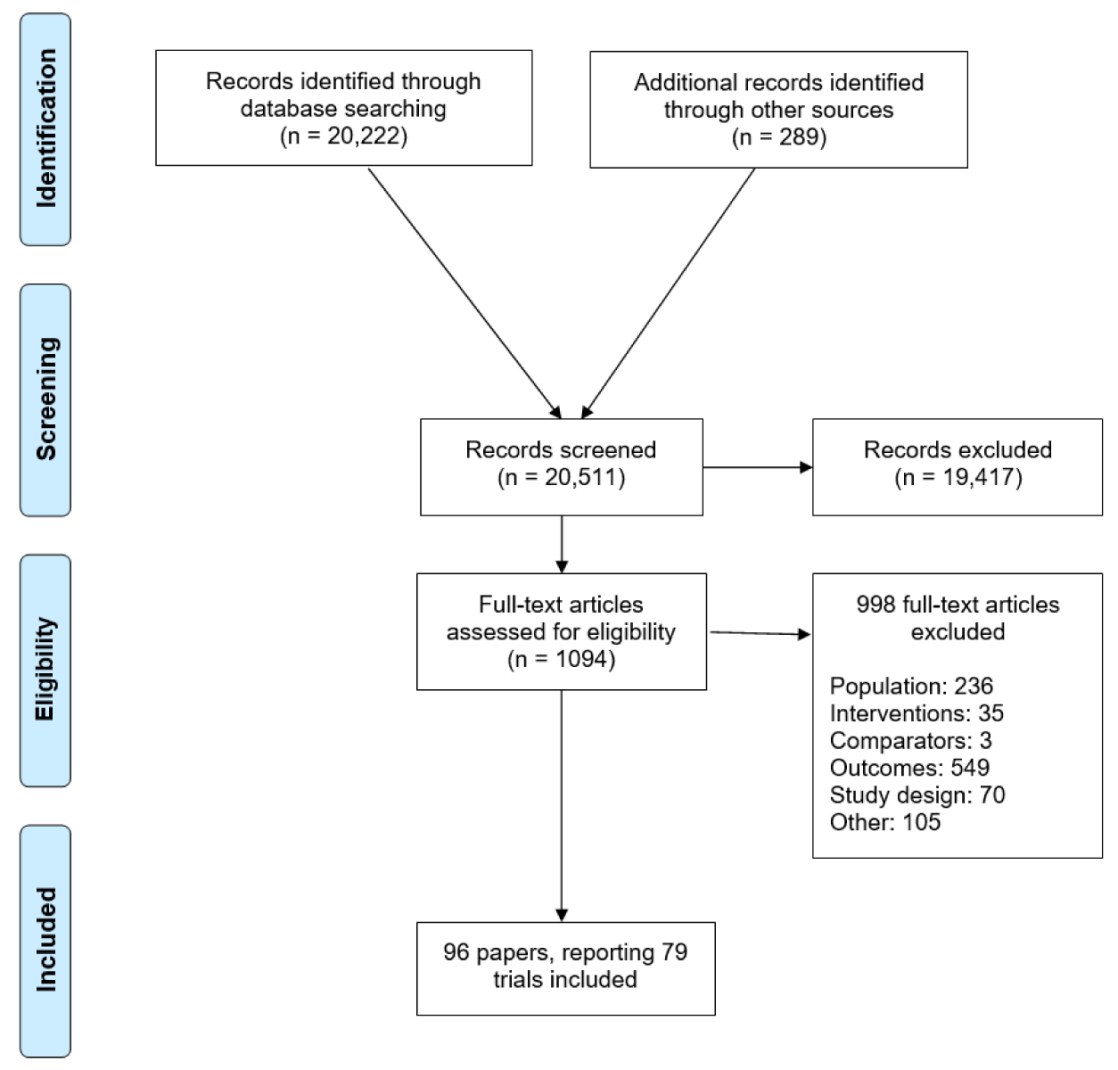
Study selection.

**Figure 2.**
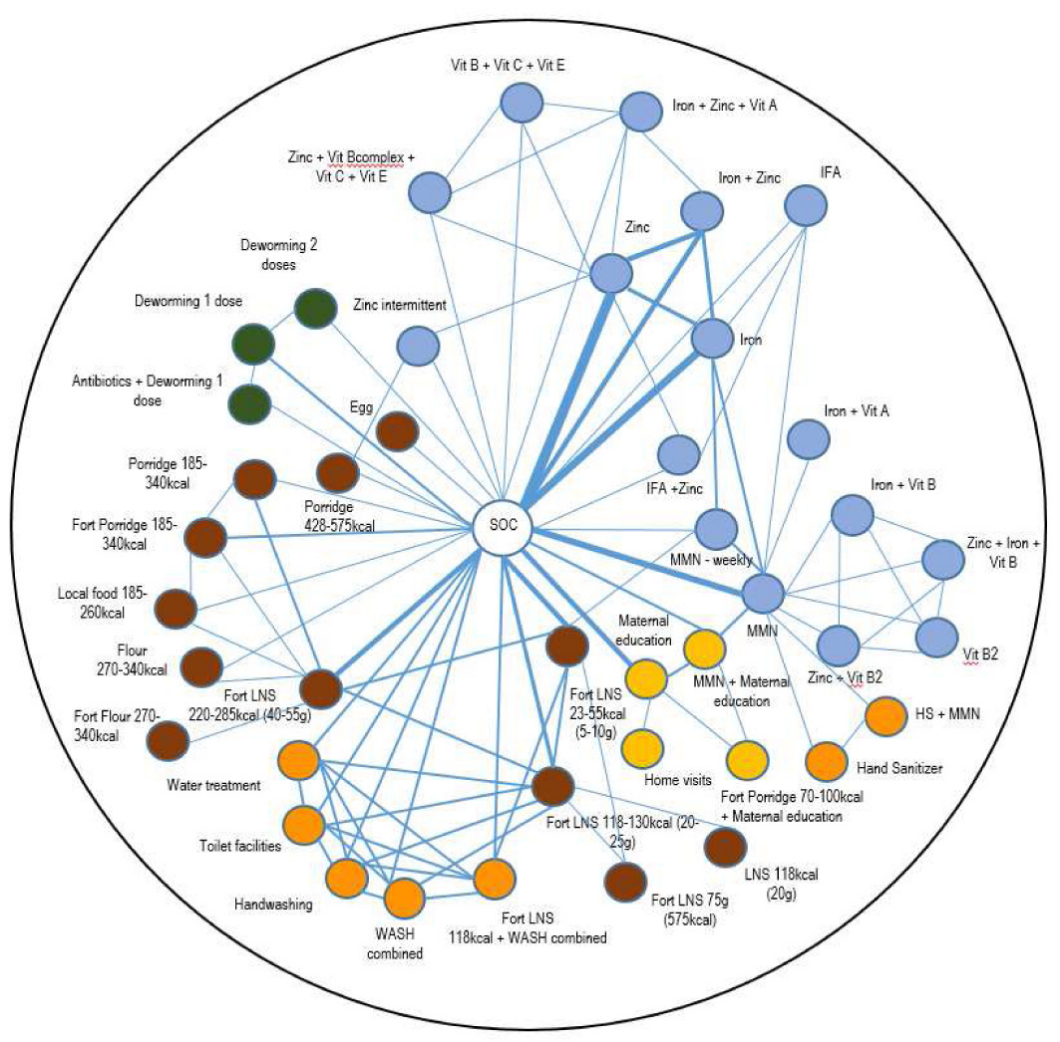
Overall network of the comparisons between interventions for children aged 6 to 24 months. Each node (circle) represents an intervention, each line represents a direct comparison between interventions, with the lines with width representing the number of trials with the direct comparisons in question (i.e. thicker width represents a direct comparison with larger numbers of trials). The different intervention domains are indicated with the following colors: blue for micronutrient supplements; brown for food supplements; yellow for education and counseling interventions; green for deworming interventions; and orange for WASH interventions. Vit. vitamin; IFA, iron and folic acid; LNS, lipid-based nutrient supplements; Fort, fortification; MMN, multiple micronutrients; HS, hand sanitizer.

A total of 20,511 abstracts was found from our database searches and hand searches of the bibliography of published reviews ( Figure 1). Of these, 1,094 studies underwent a full-text review with 96 papers reporting on 79 trials that met the inclusion criteria. The list of included studies is provided in the *Extended data* (Supplementary Table 4 for included and Table 5 for the list of excluded studies) ^18^. Trial characteristics and participant characteristics of the included studies are provided in the *Extended data*, Supplementary Tables 6 and 7 ^18^, respectively. In total, these trials comprised of 81,786 children who were randomized to 236 unique interventions ( Figure 2). Of these trials, 22 were cluster trials with 2,990 clusters (53,057 children) randomized to 80 interventions. The majority of trials were conducted in African (n = 35) and South Eastern Asian (n = 25) countries with double blinding (i.e. blinding of participants and investigators; n = 40) being the most common blinding feature. Micronutrient supplementation was the most common intervention domain studied (n = 50 trials). Only a handful of these micronutrient trials compared interventions from other domains (food supplements: n = 11 trials ^46– 56^ and maternal education: n = 2 trial ^57, 58^). There were four cluster trials on WASH (WASH Benefits Bangladesh ^59^, WASH Benefits Kenya ^60^, SHINE ^61^, and Shafique *et al.* ^62^), and these trials also included intervention arms that consisted of food supplements (i.e. LNS) or multiple micronutrients (i.e. MMN).

### Height-for-age z-score (HAZ)

**Figure 3.**
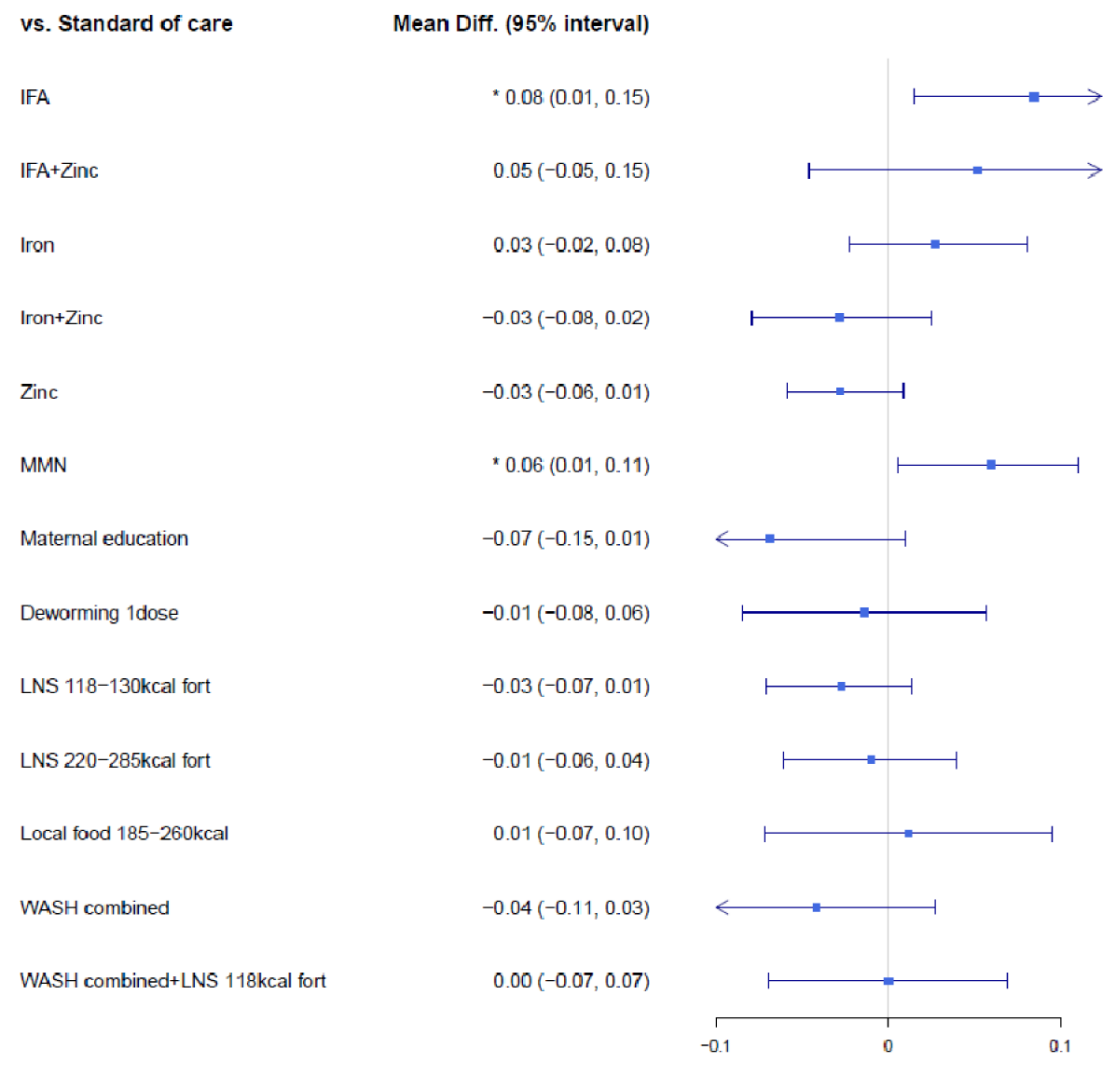
Forest plot for the effects of interventions on HAZ (mean difference), primary analysis. Vit. vitamin; IFA, iron and folic acid; LNS, lipid-based nutrient supplements; Fort, fortification; MMN, multiple micronutrients; WASH, – water treatment, toilet facilities, and handwashing. * Denotes mean difference values that do not contain the null effect of 0.

The network of evidence pertaining to the analysis of the outcome HAZ included 67 trials (69,223 children randomized to 220 intervention arms; *Extended data*, Supplementary Figure 1) ^18^. Of these, 16 were cluster trials that randomized 1,440 clusters (36,032 children) to 62 intervention arms. Key results of the primary analysis that included both cluster and non-cluster randomized clinical trials are illustrated using a forest plot ( Figure 3). Among micronutrient supplementations, iron + folic acid (IFA) (mean difference =0.08 95% CrI: 0.01, 0.15), and multiple micronutrients (MMN) (mean difference =0.06; 95% CrI: 0.01, 0.11) showed improvements in HAZ in comparison to standard-of-care. Iron (mean difference =0.03; 95% Crl: -0.02, 0.08) showed a trend towards HAZ improvement versus standard-of-care, but its credible intervals contained the null effect of 0. No food supplements showed improvements for HAZ versus standard-of-care. Similarly, no deworming interventions during the complementary feeding period or WASH interventions showed improvements in HAZ compared to standard-of-care.

### Sensitivity analysis for HAZ

The network diagram of the sensitivity analysis restricted to non-cluster randomized clinical trials for HAZ is provided in the *Extended data* (Supplementary Crosstable and Supplementary Tables and Figures) ^18^. In comparison to standard of care, IFA showed results highly consistent with the primary analysis (mean difference =0.08; 95% CrI: 0.00, 0.16). MMN, on the other hand, did not show effectiveness over standard-of-care in the sensitivity analysis, but the trend was similar to the findings from the primary analysis (mean difference =0.05; 95% CrI: -0.03, 0.12). Similar to the primary analysis, no deworming and food supplements showed improvements in HAZ in comparison to standard-of-care. No WASH interventions were available for the sensitivity analysis, as it was limited to non-cluster trials only.

### Stunting (HAZ < -2SD)

**Figure 4.**
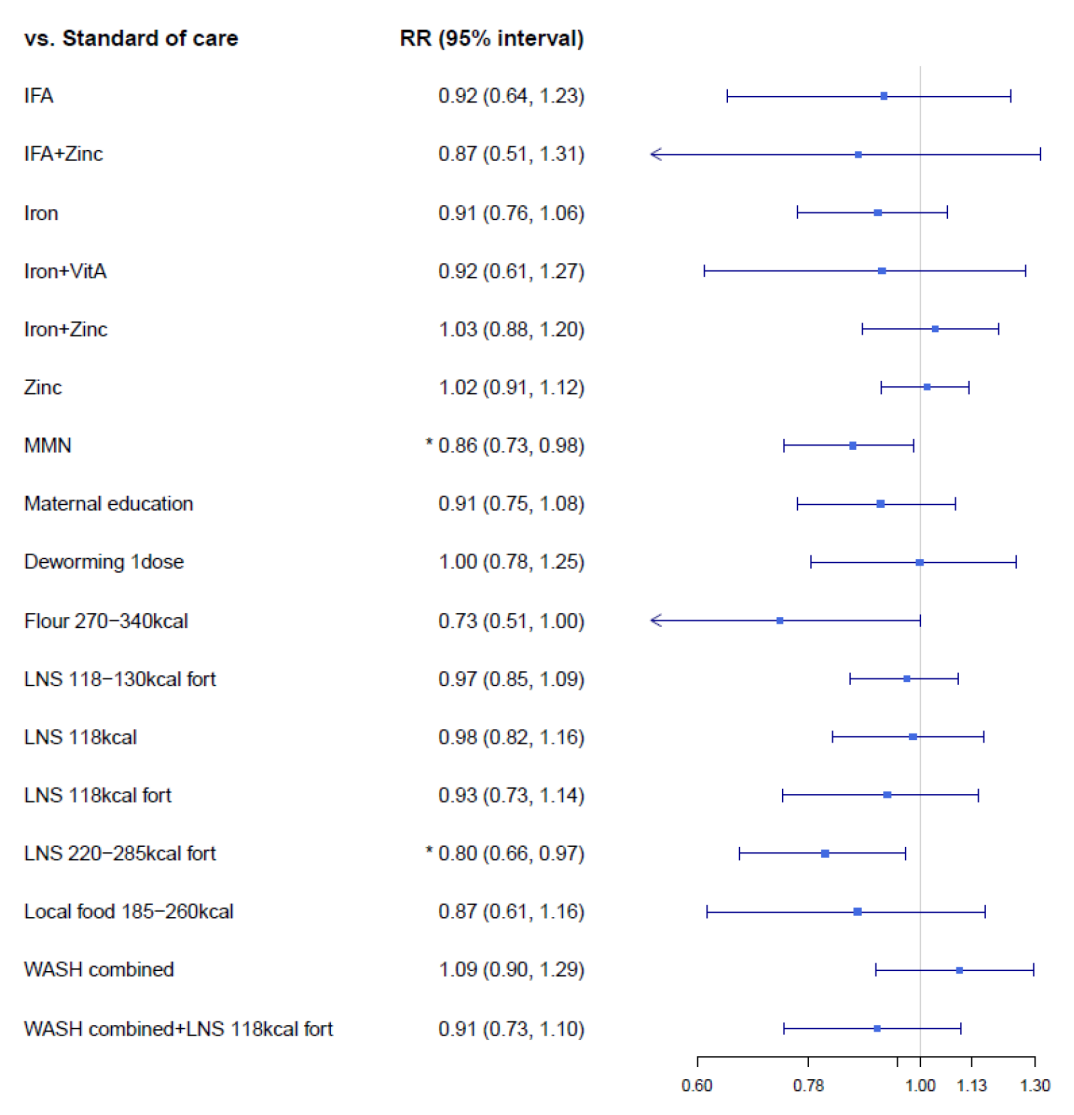
Forest plot for the effects of interventions on stunting (risk ratio), primary analysis. Vit. vitamin; IFA, iron and folic acid; LNS, lipid-based nutrient supplements; Fort, fortification; MMN, multiple micronutrients; WASH, – water treatment, toilet facilities, and handwashing. * Denotes values of risk ratio that do not contain the null effect of 1.

The network of evidence for the primary analysis of the stunting outcome consisted of 20 trials with 40,193 children randomized to 77 intervention arms ( *Extended data*, Supplementary Figure 3) ^18^. Of these, 12 were cluster trials with 1,608 clusters (33,660 children) randomized to 50 intervention arms. A forest plot for the comparative effects of interventions on stunting (RRs) is provided in Figure 4. Among micronutrient supplements, MMN (RR: 0.86, 95% CrI: 0.73, 0.98) demonstrated superiority over standard-of-care, whereas IFA (RR: 0.92, 95% CrI: 0.64, 1.23) did not reduce the risks of stunting relative to standard-of-care; however, intake of Iron (RR: 0.91; 95% Crl: 0.76, 1.06) showed trend towards reducing risks for stunting. For food supplements, fortified lipid-based nutrient supplements (LNS) containing 220–285 kcal (RR: 0.80, 95% CrI: 0.66, 0.97) and flour containing 270 – 340 kcal (RR: 0.73, 95% CrI: 0.51, 1.00) showed reduced risks of stunting versus standard-of-care. Among other intervention domains, compared to standard-of-care, Maternal education also showed a trend towards decreasing the risks of stunting (RR: 0.91, 95% CrI: 0.75, 1.08); but no deworming or WASH interventions showed reduced risks for stunting except for WASH combined + fortified LNS containing 118 kilocalories that had showed a trend towards reducing the risks of stunting (RR: 0.91, 95% CrI: 0.73, 1.10).

### Sensitivity analysis for stunting

The network diagram of the sensitivity analysis for stunting is provided in the *Extended data* (Supplementary Figure 4) ^18^. The results of the sensitivity analyses are provided in the *Extended data*, Supplementary Cross-table file (tab: “Sensitivity, Stunting”) ^18^. We found several interventions that showed either reduced risks for stunting or trends towards reduced risks. For instance, relative to standard-of-care, MMN (RR: 0.78; 95% CrI: 0.59, 1.00) and fortified LNS 220–285 kcal (RR: 0.80; 95% Crl: 0.62, 1.00) showed reduced risks for stunting. Similarly, iron (RR: 0.89, 95% CrI: 0.74, 1.06) and flour 270–340 kcal (RR: 0.71; 95% Crl: 0.48, 1.02), showed trends towards reducing the risks of stunting versus standard-of-care, but their Crls contained the null effect of 1. No deworming interventions showed reduced risks for stunting over standard-of-care. Similar to the sensitivity analysis for HAZ, no WASH interventions were available.

## Discussion

In this study, systematic literature review and network meta-analysis were used to determine the comparative effectiveness of interventions for linear growth under the domains of micronutrients, food supplements, deworming, maternal education, and WASH interventions for LMIC-based children in the age group of 6 to 24 months. During the complementary feeding period life stage, micronutrient supplements such as IFA and MMN showed improvements for HAZ compared to standard-of-care, with iron showing some trends towards improved HAZ. Deworming, maternal education, food supplements, and WASH interventions, on the other hand, did not show improvements in HAZ versus standard-of-care. For stunting, food supplements of fortified LNS 220–280 kcal and flour 270–340 kcal showed reduced risks of stunting, with other interventions such as iron, MMN, and MMN combined with maternal education, demonstrating trends towards reduced stunting risks, in comparison to standard-of-care.

 The key strengths of this study were the consideration of multiple intervention domains and the use of network meta-analysis. The approach undertaken for this study differed from previous reviews ( Table 1) that have had limited scopes of single intervention or single intervention domains; these reviews have all used pairwise meta-analysis, thus being limited to trials and interventions that have only been directly compared to one another. The use of network meta-analysis allowed for consideration of a broader evidence base to estimate the comparative effectiveness of multiple interventions under multiple treatment domains. By incorporating statistical adjustments for clustering effects, this study was able to incorporate cluster randomized clinical trials that mostly did not report information on clustering effects (i.e. ICC or design effects) into the statistical analyses.

Nonetheless, the existing evidence base limited our analyses. The majority of the randomized clinical trial evidence base was confined to the micronutrient supplementation domain (n = 50), and the evidence base for intervention domains for food supplements, deworming, maternal education, and WASH being limited. This imbalance in intervention class could partly be explained by the narrow bounds in the population criterion (i.e. the age criteria of 6 to 24 months) of our PICOS criteria. For instance, there were a number of trials that recruited children using a wider age eligibility criterion (e.g. 6 months up to 5 years of age) that encompassed children in the complementary feeding life stage, but these trials were excluded since growth rates and determinants of children older than 24 months are different than children in the complementary feeding period age group. While this age restriction limited the number of eligible trials for our analysis, it is important to note that the population criteria was determined a priori before the screening was initiated for this systematic review since we recognized that growth determinants and rates can vary substantially for children between these ages ^63^. Another limitation related to our categorisation of interventions is that we combined interventions into broad categories to assist with interpretation and acknowledge that a different approach to categorisation might have altered the results.

This study has shown that the existing evidence base on interventions aimed to improve linear growth in children during the complementary feeding period is limited and inconsistent. Generally, investigation of interventions outside of the domain of micronutrient supplements was limited. There were only two trials on deworming ^64, 65^ and four WASH trials ^59– 62^ reporting on linear growth outcomes in our analyses. There were eight trials under the maternal education domains ^57, 58, 62, 66– 70^, but the components and the delivery of these educational interventions varied considerably between these trials. For food supplement trials, poor adherence and household food insecurity that may promote family sharing could influence why these interventions did not show improvements in linear growth. Moreover, the food supplements in these trials were all based on a single type of food, so children may have refused to consume as they have become tired of consuming same type of food over time. There were no trials that investigated nutritional strategies that aimed to improve dietary diversity in order to improve linear growth in children during this life stage, nor were there any trials that aimed to address household food insecurity. It is also important to note that there was randomness in the data as well as substantial heterogeneity observed in the duration of the interventions and the timing of outcome assessments that can be attributed to the variation in impact on HAZ and stunting.

A previous report from Bangladesh has shown that the taste of LNS is generally acceptable, and at least in shorter-term, adherence to LNS was high and sharing of these food supplements could be minimized in the household ^71^. However, acceptability and adherence to LNS in other settings are not clear; the long-term acceptability and adherence to LNS or other types of food supplements that consist of a single food on daily basis over long-term are also questionable. Additionally, our analysis did not show that supplementing children with high caloric food supplements result in improved linear growth when compared to standard of care or to other lower caloric food supplements. Aside from previously described issues associated with tolerability of nutritional supplementation that may be exacerbated by high caloric formulations, it is possible that in households with food insecurity, caregivers may choose to share these supplements with other children or members of the household who are not enrolled in the trial. Understanding compliance and the influence that this may have on our analyses was not possible, as we found that compliance was usually not measured or reported in the included food supplement trials ^72– 76^.

Our findings identified several directions for future research. There is a need to combine interventions across multiple domains as a package. Instead of singling out interventions from one domain, there is a need for comparisons between different packaged interventions because a combined set of interventions will likely result in the greatest improvements in linear growth. Strategies should consider local contexts and challenges. The feasibility of conducting trials that incorporate food supplement strategies aimed to improve dietary diversity and address household food insecurities is undoubtedly difficult. However, it is important to recognize these factors will be important for long-term acceptability and adherence to food interventions, and interventional strategies that incorporate diverse local foods will have higher acceptability and adherence in the long run. Trials with longer follow-up are also needed, as the median follow-up among the trials included for this review was only 6 months. Lastly, there is a need for more innovative trial approaches ^77^. The majority of the trials identified for this review used a conventional trial approach with a fixed sample size design, where the assessment of interventions occurred only after the number of participants recruited reached the sample size target. It is important to recognize that such approach can be inefficient, and adopting adaptive trial design approaches that allow for pre-specified modifications, with the decision being made based on accumulating trial data may improve both the efficiencies of the trial evaluation in this avenue of research ^78^.

## Data availability

### Underlying data

All data underlying the results are available as part of the article and no additional source data are required.

### Extended data

Open Science Framework: Interventions to improve linear growth during complementary feeding period for children aged 6–24 months living in low- and middle-income countries: a systematic review and network meta-analysis. https://doi.org/10.17605/OSF.IO/DTZK7 ^18^.

This project contains the following extended data:

- Complementary feeding period NMA - Supplementary Tables and Figures – v3.0
  - Appendix 1. Literature search. (Contains Supplementary Tables 1–3.)
  - Appendix 2. List of included and excluded studies after full-text review. (Contains Supplementary Tables 4 and 5.)
  - Appendix 3. Details of the evidence base. (Contains Supplementary Tables 6 and 7.)
  - Appendix 4. Network for HAZ (Contains Supplementary Figures 1 and 2.)
  - Appendix 5. Network for stunting outcome. (Contains Supplementary Figures 3–9.)
- Complementary feeding period NMA - Supplementary Crosstable - v1.0

### Reporting Guidelines

Open Science Framework: PRISMA checklist for “Interventions to improve linear growth during complementary feeding period for children aged 6–24 months living in low- and middle-income countries: a systematic review and network meta-analysis.” https://doi.org/10.17605/OSF.IO/DTZK7 ^18^.

Data are available under the terms of the Creative Commons Attribution 4.0 International license (CC-BY 4.0).

## References

[1] Suchdev PS Boivin MJ Forsyth BW: Assessment of Neurodevelopment, Nutrition, and Inflammation From Fetal Life to Adolescence in Low-Resource Settings. *Pediatrics.* 2017;139(Suppl 1):S23–S37. 10.1542/peds.2016-2828E 28562246

[2] Black RE Victora CG Walker SP: Maternal and child undernutrition and overweight in low-income and middle-income countries. *Lancet.* 2013;382(9890):427–51. 10.1016/S0140-6736(13)60937-X 23746772

[3] de Onis M Branca F: Childhood stunting: a global perspective. *Matern Child Nutr.* 2016;12 Suppl 1:12–26. 10.1111/mcn.12231 27187907 PMC5084763

[4] Stewart CP Iannotti L Dewey KG: Contextualising complementary feeding in a broader framework for stunting prevention. *Matern Child Nutr.* 2013;9 Suppl 2:27–45. 10.1111/mcn.12088 24074316 PMC6860787

[5] Mendez MA Adair LS: Severity and timing of stunting in the first two years of life affect performance on cognitive tests in late childhood. *J Nutr.* 1999;129(8):1555–62. 10.1093/jn/129.8.1555 10419990

[6] Dewey KG Mayers DR: Early child growth: how do nutrition and infection interact? *Matern Child Nutr.* 2011;7 Suppl 3:129–42. 10.1111/j.1740-8709.2011.00357.x 21929641 PMC6860756

[7] Perkins JM Subramanian SV Davey Smith G: Adult height, nutrition, and population health. *Nutr Rev.* 2016;74(3):149–65. 10.1093/nutrit/nuv105 26928678 PMC4892290

[8] Kolčić I: Double burden of malnutrition: A silent driver of double burden of disease in low- and middle-income countries. *J Glob Health.* 2012;2(2):020303. 10.7189/jogh.02.020303 23289074 PMC3529312

[9] McGovern ME Krishna A Aguayo VM: A review of the evidence linking child stunting to economic outcomes. *Int J Epidemiol.* 2017;46(4):1171–91. 10.1093/ije/dyx017 28379434 PMC5837457

[10] Mills EJ Thorlund K Ioannidis JP: Demystifying trial networks and network meta-analysis. *BMJ.* 2013;346:f2914. 10.1136/bmj.f2914 23674332

[11] Kanters S Ford N Druyts E: Use of network meta-analysis in clinical guidelines. *Bull World Health Organ.* 2016;94(10):782. 10.2471/BLT.16.174326 27843171 PMC5043215

[12] Kanters S Vitoria M Doherty M: Comparative efficacy and safety of first-line antiretroviral therapy for the treatment of HIV infection: a systematic review and network meta-analysis. *Lancet HIV.* 2016;3(11):e510–e20. 10.1016/S2352-3018(16)30091-1 27658869

[13] World Health Organization: Consolidated guidelines on HIV prevention, diagnosis, treatment and care for key populations 2016 update. World Health Organization;2016 Reference Source 27559558

[14] World Health Organization: Guidelines for the screening, care and treatment of persons with chronic hepatitis C infection. World Health Organization;2016 Reference Source 27227200

[15] Hutton B Salanti G Caldwell DM: The PRISMA extension statement for reporting of systematic reviews incorporating network meta-analyses of health care interventions: checklist and explanations. *Ann Intern Med.* 2015;162(11):777–84. 10.7326/M14-2385 26030634

[16] Bhutta ZA Das JK Rizvi A: Evidence-based interventions for improvement of maternal and child nutrition: what can be done and at what cost? *Lancet.* 2013;382(9890):452–77. 10.1016/S0140-6736(13)60996-4 23746776

[17] Ruel MT Alderman H, Maternal and Child Nutrition Study Group: Nutrition-sensitive interventions and programmes: how can they help to accelerate progress in improving maternal and child nutrition? *Lancet.* 2013;382(9891):536–51. 10.1016/S0140-6736(13)60843-0 23746780

[18] Park JJ Harari O Siden E: Interventions to improve linear growth during complementary feeding period for children aged 6-24 months living in low- and middle-income countries: a systematic review and network meta-analysis.2019 10.17605/OSF.IO/DTZK7 PMC7096089 32259047

[19] World Health Organization: Child growth standards: length/height-for-age. (accessed Aug 15 2017).2015 Reference Source

[20] Dangour AD Watson L Cumming O: Interventions to improve water quality and supply, sanitation and hygiene practices, and their effects on the nutritional status of children. *Cochrane Database Syst Rev.* 2013; (8):CD009382. 10.1002/14651858.CD009382.pub2 23904195 PMC11608819

[21] Darlow BA Graham PJ Rojas-Reyes MX: Vitamin A supplementation to prevent mortality and short- and long-term morbidity in very low birth weight infants. *Cochrane Database Syst Rev.* 2016;2016(8):CD000501. 10.1002/14651858.CD000501.pub4 27552058 PMC7038719

[22] Das JK Salam RA Kumar R: Micronutrient fortification of food and its impact on woman and child health: a systematic review. *Syst Rev.* 2013;2:67. 10.1186/2046-4053-2-67 23971426 PMC3765883

[23] De-Regil LM Jefferds ME Sylvetsky AC: Intermittent iron supplementation for improving nutrition and development in children under 12 years of age. *Cochrane Database Syst Rev.* 2011;2011(12):CD009085. 10.1002/14651858.CD009085.pub2 22161444 PMC4547491

[24] De-Regil LM Suchdev PS Vist GE: Home fortification of foods with multiple micronutrient powders for health and nutrition in children under two years of age (Review). *Evid Based Child Health.* 2013;8(1):112–201. 10.1002/ebch.1895 23878126

[25] Devakumar D Fall CH Sachdev HS: Maternal antenatal multiple micronutrient supplementation for long-term health benefits in children: a systematic review and meta-analysis. *BMC Med.* 2016;14:90. 10.1186/s12916-016-0633-3 27306908 PMC4910255

[26] Gaffey MF Wazny K Bassani DG: Dietary management of childhood diarrhea in low- and middle-income countries: a systematic review. *BMC Public Health.* 2013;13 Suppl 3:S17. 10.1186/1471-2458-13-S3-S17 24564685 PMC3847348

[27] Gough EK Moodie EE Prendergast AJ: The impact of antibiotics on growth in children in low and middle income countries: systematic review and meta-analysis of randomised controlled trials. *BMJ.* 2014;348:g2267. 10.1136/bmj.g2267 24735883 PMC3988318

[28] Imdad A Bhutta ZA: Effect of preventive zinc supplementation on linear growth in children under 5 years of age in developing countries: a meta-analysis of studies for input to the lives saved tool. *BMC Public Health.* 2011;11 Suppl 3:S22. 10.1186/1471-2458-11-S3-S22 21501440 PMC3231896

[29] Imdad A Mayo-Wilson E Herzer K: Vitamin A supplementation for preventing morbidity and mortality in children from six months to five years of age. *Cochrane Database Syst Rev.* 2017;3(3):CD008524. 10.1002/14651858.CD008524.pub3 28282701 PMC6464706

[30] Kristjansson E Francis DK Liberato S: Food supplementation for improving the physical and psychosocial health of socio-economically disadvantaged children aged three months to five years. *Cochrane Database Syst Rev.* 2015;2015(3):CD009924. 10.1002/14651858.CD009924.pub2 25739460 PMC6885042

[31] Lassi ZS Das JK Zahid G: Impact of education and provision of complementary feeding on growth and morbidity in children less than 2 years of age in developing countries: a systematic review. *BMC Public Health.* 2013;13(Suppl 3): S13. 10.1186/1471-2458-13-S3-S13 24564534 PMC3847349

[32] Matsungo TM Kruger HS Smuts CM: Lipid-based nutrient supplements and linear growth in children under 2 years: a review. *Proc Nutr Soc.* 2017;76(4):580–8. 10.1017/S0029665117000283 28285607

[33] Mayo-Wilson E Junior JA Imdad A: Zinc supplementation for preventing mortality, morbidity, and growth failure in children aged 6 months to 12 years of age. *Cochrane Database Syst Rev.* 2014; (5):CD009384. 10.1002/14651858.CD009384.pub2 24826920

[34] Pasricha SR Hayes E Kalumba K: Effect of daily iron supplementation on health in children aged 4–23 months: a systematic review and meta-analysis of randomised controlled trials. *Lancet Glob Health.* 2013;1(2):e77–e86. 10.1016/S2214-109X(13)70046-9 25104162

[35] Petry N Olofin I Boy E: The Effect of Low Dose Iron and Zinc Intake on Child Micronutrient Status and Development during the First 1000 Days of Life: A Systematic Review and Meta-Analysis. *Nutrients.* 2016;8(12): pii: E773. 10.3390/nu8120773 27916873 PMC5188428

[36] Salam RA MacPhail C Das JK: Effectiveness of Micronutrient Powders (MNP) in women and children. *BMC Public Health.* 2013;13(Suppl 3):S22. 24564207 10.1186/1471-2458-13-S3-S22 PMC3847468

[37] Sguassero Y de Onis M Bonotti AM: Community-based supplementary feeding for promoting the growth of children under five years of age in low and middle income countries. *Cochrane Database Syst Rev.* 2012; (6):CD005039. 10.1002/14651858.CD005039.pub3 22696347 PMC8078353

[38] Taylor-Robinson DC Maayan N Soares-Weiser K: Deworming drugs for soil-transmitted intestinal worms in children: effects on nutritional indicators, haemoglobin, and school performance. *Cochrane Database Syst Rev.* 2015; (7):CD000371. 10.1002/14651858.CD000371.pub6 26202783 PMC4523932

[39] R Development Core Team: R: A Language Environment for Statistical Computing. In:Team RC editor. Vienna, Austria: R Foundation for Statistical Computing;2017 Reference Source

[40] Sturtz S Ligges U Gelman A: R2OpenBUGS: a package for running OpenBUGS from R. *J Stat Softw.* 2005;12(3):1–16. Reference Source

[41] Dias S Sutton AJ Ades AE: Evidence synthesis for decision making 2: a generalized linear modeling framework for pairwise and network meta-analysis of randomized controlled trials. *Med Decis Making.* 2013;33(5):607–17. 10.1177/0272989X12458724 23104435 PMC3704203

[42] Rhodes KM Turner RM White IR: Implementing informative priors for heterogeneity in meta-analysis using meta-regression and pseudo data. *Stat Med.* 2016;35(29):5495–511. 10.1002/sim.7090 27577523 PMC5111594

[43] Turner RM Jackson D Wei Y: Predictive distributions for between-study heterogeneity and simple methods for their application in Bayesian meta-analysis. *Stat Med.* 2015;34(6):984–98. 10.1002/sim.6381 25475839 PMC4383649

[44] Uhlmann L Jensen K Kieser M: Bayesian network meta-analysis for cluster randomized trials with binary outcomes. *Res Synth Methods.* 2017;8(2):236–50. 10.1002/jrsm.1210 27390267

[45] Higgins JP Thomas J Chandler J: Cochrane Handbook for Systematic Reviews of Interventions version 6.0.Cochrane Reviews;2019 Reference Source

[46] Skau JK Touch B Chhoun C: Effects of animal source food and micronutrient fortification in complementary food products on body composition, iron status, and linear growth: a randomized trial in Cambodia. *Am J Clin Nutr.* 2015;101(4):742–51. 10.3945/ajcn.114.084889 25833972

[47] Iannotti LL Dulience SJ Green J: Linear growth increased in young children in an urban slum of Haiti: a randomized controlled trial of a lipid-based nutrient supplement. *Am J Clin Nutr.* 2014;99(1):198–208. 10.3945/ajcn.113.063883 24225356 PMC3862455

[48] Rivera JA González-Cossío T Flores M: Multiple micronutrient supplementation increases the growth of Mexican infants. *Am J Clin Nutr.* 2001;74(5): 657–63. 10.1093/ajcn/74.5.657 11684535

[49] Krebs NF Mazariegos M Chomba E: Randomized controlled trial of meat compared with multimicronutrient-fortified cereal in infants and toddlers with high stunting rates in diverse settings. *Am J Clin Nutr.* 2012;96(4):840–7. 10.3945/ajcn.112.041962 22952176 PMC3441111

[50] Phu PV Hoan NV Salvignol B: A six-month intervention with two different types of micronutrient-fortified complementary foods had distinct short- and long-term effects on linear and ponderal growth of Vietnamese infants. *J Nutr.* 2012;142(9):1735–40. 10.3945/jn.111.154211 22810985

[51] Dewey KG Mridha MK Matias SL: Lipid-based nutrient supplementation in the first 1000 d improves child growth in Bangladesh: a cluster-randomized effectiveness trial. *Am J Clin Nutr.* 2017;105(4):944–57. 10.3945/ajcn.116.147942 28275125

[52] Phuka JC Maleta K Thakwalakwa C: Postintervention growth of Malawian children who received 12-mo dietary complementation with a lipid-based nutrient supplement or maize-soy flour. *Am J Clin Nutr.* 2009;89(1):382–90. 10.3945/ajcn.2008.26483 19056572

[53] Jones KDJ Ali R Khasira MA: Ready-to-use therapeutic food with elevated n-3 polyunsaturated fatty acid content, with or without fish oil, to treat severe acute malnutrition: a randomized controlled trial. *BMC Med.* 2015;13(1):93. 10.1186/s12916-015-0315-6 25902844 PMC4407555

[54] Phuka JC Gladstone M Maleta K: Developmental outcomes among 18-month-old Malawians after a year of complementary feeding with lipid-based nutrient supplements or corn-soy flour. *Matern Child Nutr.* 2012;8(2):239–48. 10.1111/j.1740-8709.2011.00294.x 21342456 PMC6860816

[55] Olney DK Leroy J Bliznashka L: *PROCOMIDA*, a Food-Assisted Maternal and Child Health and Nutrition Program, Reduces Child Stunting in Guatemala: A Cluster-Randomized Controlled Intervention Trial. *J Nutr.* 2018;148(9):1493–505. 10.1093/jn/nxy138 30184223 PMC6118165

[56] Smuts CM Matsungo TM Malan L: Effect of small-quantity lipid-based nutrient supplements on growth, psychomotor development, iron status, and morbidity among 6- to 12-mo-old infants in South Africa: a randomized controlled trial. *Am J Clin Nutr.* 2019;109(1):55–68. 10.1093/ajcn/nqy282 30649163 PMC6358035

[57] Jack SJ Ou K Chea M: Effect of micronutrient sprinkles on reducing anemia: a cluster-randomized effectiveness trial. *Arch Pediatr Adolesc Med.* 2012;166(9):842–50. 10.1001/archpediatrics.2012.1003 22801933

[58] Fink G Levenson R Tembo S: Home- and community-based growth monitoring to reduce early life growth faltering: an open-label, cluster-randomized controlled trial. *Am J Clin Nutr.* 2017;106(4):1070–7. 10.3945/ajcn.117.157545 28835364 PMC5611784

[59] Luby SP Rahman M Arnold BF: Effects of water quality, sanitation, handwashing, and nutritional interventions on diarrhoea and child growth in rural Bangladesh: a cluster randomised controlled trial. *Lancet Glob Health.* 2018;6(3):e302–e315. 10.1016/S2214-109X(17)30490-4 29396217 PMC5809718

[60] Null C Stewart CP Pickering AJ: Effects of water quality, sanitation, handwashing, and nutritional interventions on diarrhoea and child growth in rural Kenya: a cluster-randomised controlled trial. *Lancet Glob Health.* 2018;6(3):e316–e329. 10.1016/S2214-109X(18)30005-6 29396219 PMC5809717

[61] Humphrey JH Mbuya MNN Ntozini R: Independent and combined effects of improved water, sanitation, and hygiene, and improved complementary feeding, on child stunting and anaemia in rural Zimbabwe: a cluster-randomised trial. *Lancet Glob Health.* 2019;7(1):e132–e147. 10.1016/S2214-109X(18)30374-7 30554749 PMC6293965

[62] Shafique S Sellen DW Lou W: Mineral- and vitamin-enhanced micronutrient powder reduces stunting in full-term low-birth-weight infants receiving nutrition, health, and hygiene education: a 2 x 2 factorial, cluster-randomized trial in Bangladesh. *Am J Clin Nutr.* 2016;103(5):1357–69. 10.3945/ajcn.115.117770 27053383

[63] Gluckman PD Hanson MA Cooper C: Effect of in utero and early-life conditions on adult health and disease. *N Engl J Med.* 2008;359(1):61–73. 10.1056/NEJMra0708473 18596274 PMC3923653

[64] Goto R Mascie-Taylor CG Lunn PG: Impact of anti-Giardia and anthelminthic treatment on infant growth and intestinal permeability in rural Bangladesh: a randomised double-blind controlled study. *Trans R Soc Trop Med Hyg.* 2009;103(5):520–9. 10.1016/j.trstmh.2008.07.020 18789466

[65] Joseph SA Casapia M Montresor A: The Effect of Deworming on Growth in One-Year-Old Children Living in a Soil-Transmitted Helminth-Endemic Area of Peru: A Randomized Controlled Trial. *PLoS Negl Trop Dis.* 2015;9(10):e0004020. 10.1371/journal.pntd.0004020 26426270 PMC4591279

[66] Muhoozi GKM Atukunda P Diep LM: Nutrition, hygiene, and stimulation education to improve growth, cognitive, language, and motor development among infants in Uganda: A cluster‐randomized trial. *Matern Child Nutr.* 2018;14(2):e12527. 10.1111/mcn.12527 28925580 PMC6866193

[67] Nair N Tripathy P Sachdev H: Effect of participatory women's groups and counselling through home visits on children's linear growth in rural eastern India(CARING trial): a cluster-randomised controlled trial. *Lancet Glob Health.* 2017;5(10):e1004–e1016. 10.1016/S2214-109X(17)30339-X 28911749 PMC5640793

[68] Nikièma L Huybregts L Martin-Prevel Y: Effectiveness of facility-based personalized maternal nutrition counseling in improving child growth and morbidity up to 18 months: A cluster-randomized controlled trial in rural Burkina Faso. *PLoS One.* 2017;12(5):e0177839. 10.1371/journal.pone.0177839 28542391 PMC5444625

[69] Saleem AF Mahmud S Baig-Ansari N: Impact of maternal education about complementary feeding on their infants' nutritional outcomes in low- and middle-income households: a community-based randomized interventional study in Karachi, Pakistan. *J Health Popul Nutr.* 2014;32(4):623–33. 25895196 PMC4438693

[70] Osei AK Pandey P Spiro D: Adding multiple micronutrient powders to a homestead food production programme yields marginally significant benefit on anaemia reduction among young children in Nepal. *Matern Child Nutr.* 2015;11 Suppl 4:188–202. 10.1111/mcn.12173 25682798 PMC6860240

[71] Mridha M Chaparro C Matias S: Acceptability of lipid-based nutrient supplements and micronutrient powders among pregnant and lactating women and infants and young children in Bangladesh and their perceptions about malnutrition and nutrient supplements. Washington, DC: FHI.2012;360 Reference Source

[72] Cohen RJ Brown KH Canahuati J: Determinants of Growth From Birth to 12 Months Among Breast-Fed Honduran Infants in Relation to Age of Introduction of Complementary Foods. *Pediatrics.* 1995;96(3 pt 1): 504–10. 7651785

[73] Thakwalakwa C Ashorn P Phuka J: A lipid-based nutrient supplement but not corn-soy blend modestly increases weight gain among 6 to 18 month-old moderately underweight children in rural Malawi. *J Nutr.* 2010;140(11):2008–13. 10.3945/jn.110.122499 20861218

[74] Oelofse A Van Raaij JM Benade AJ: The effect of a micronutrient-fortified complementary food on micronutrient status, growth and development of 6 to 12 month-old disadvantaged urban South African infants. *Int J Food Sci Nutr.* 2003;54(5):399–407. 10.1080/0963748031000092161 12907410

[75] Lartey A Manu A Brown KH: A randomized, community-based trial of the effects of improved, centrally processed complementary foods on growth and micronutrient status of Ghanaian infants from 6 to 12 mo of age. *Am J Clin Nutr.* 1999;70(3):391–404. 10.1093/ajcn/70.3.391 10479202

[76] Kuusipalo H Maleta K Briend A: Growth and change in blood haemoglobin concentration among underweight Malawian infants receiving fortified spreads for 12 weeks: a preliminary trial. *J Pediatr Gastroenterol Nutr.* 2006;43(4):525–32. 10.1097/01.mpg.0000235981.26700.d3 17033530

[77] Park JJ Thorlund K Mills EJ: Critical concepts in adaptive clinical trials. *Clin Epidemiol.* 2018;10:343–51. 10.2147/CLEP.S156708 29606891 PMC5868584

[78] Thorlund K Haggstrom J Park JJ: Key design considerations for adaptive clinical trials: a primer for clinicians. *BMJ.* 2018;360:k698. 10.1136/bmj.k698 29519932 PMC5842365

