# Supplementary material for: Interventions to improve linear growth during complementary feeding period for children aged 6-24 months living in low- and middle-income countries: a systematic review and network meta-analysis
Source: Gates Open Res. 2020 Sep 24;3:1660. Originally published 2019 Nov 14. [Version 2] doi: 10.12688/gatesopenres.13083.2 (PMC7096089; doi:10.12688/gatesopenres.13083.2)
Supplement: Peer review history

# Peer review: Reviewer response for version 2

**DOI:** 10.21956/gatesopenres.14383.r29668

**Published:** 2020-09-24

**Authors:** Judd L. Walson

**Affiliations:** 1 Department of Global Health, University of Washington, Seattle, WA, USA

The authors have responded clearly to my initial comments.

Are the rationale for, and objectives of, the Systematic Review clearly stated?

Yes

Is the statistical analysis and its interpretation appropriate?

Partly

Are sufficient details of the methods and analysis provided to allow replication by others?

Yes

Are the conclusions drawn adequately supported by the results presented in the review?

Partly

Reviewer Expertise:

infectious disease, nutrition, trials in LMIC settings

I confirm that I have read this submission and believe that I have an appropriate level of expertise to confirm that it is of an acceptable scientific standard.

# Peer review: Reviewer response for version 1

**DOI:** 10.21956/gatesopenres.14225.r28444

**Published:** 2020-03-19

**Authors:** Kenneth Maleta

**Affiliations:** 1 School of Public Health and Family Medicine, University of Malawi, Blantyre, Malawi

The authors tackle an otherwise vexing question of the limited success of interventions to address linear growth faltering and stuntedness by employing network analysis which allowed for assessing multiple interventions and their comparative effects on the outcomes. The statistical approach is very well justified and described. Overall the paper is very well written save for a few areas where the authors need to clarify or speculate reasons for their findings:

1. The authors should explain why they think certain interventions had an impact on HAZ index (HAZ<-2.0) but not on actual HAZ.
2. Considering that the interventions were of variable duration, did the authors explore sensitivity analyses that limited analyses to intervention domains that were implemented for comparable periods of time (i.e. within each domain)? If not possible then some explanation and concession on how this could have affected the results should be included in the write up.
3. It is commonly accepted that interventions' effects vary depending on context. In the realm of this analysis it seems it is not possible to include effect modification analysis and if that is true, the discussion should highlight the impact lack of such analysis may have had on the findings.
4. I am not entirely clear on the definition of standard of care. In Table 2 this is defined as placebo/no intervention/standard of care/or monotherapy or combination therapy that can be used for indirect comparison. This seems too broad and heterogeneous. Did the authors try narrowing this down further to the placebo/no intervention and rest? How did this very broad categorization affect the findings?

Are the rationale for, and objectives of, the Systematic Review clearly stated?

Yes

Is the statistical analysis and its interpretation appropriate?

Yes

Are sufficient details of the methods and analysis provided to allow replication by others?

Yes

Are the conclusions drawn adequately supported by the results presented in the review?

Partly

Reviewer Expertise:

Nutrition, Epidemiology, Clinical trials in LMIC.

I confirm that I have read this submission and believe that I have an appropriate level of expertise to confirm that it is of an acceptable scientific standard.

# Response

**Published:** 2020-08-21

**Authors:** Edward Mills

Dear Dr. Maleta:

Thank you for your thorough review of our manuscript. Our responses to your recommendations and comments are marked with bullets below.  

Sincerely,

Edward J. Mills

The authors should explain why they think certain interventions had an impact on HAZ index (HAZ<-2.0) but not on actual HAZ.

 

- *Thank you for your comment. The observed differences between the impact on HAZ and stunting can be attributed* *to randomness in the data as well as substantial heterogeneity observed in the duration of the interventions and the timing of outcome assessments. We have amended the discussion section to include this as a limitation. *

 

Considering that the interventions were of variable duration, did the authors explore sensitivity analyses that limited analyses to intervention domains that were implemented for comparable periods of time (i.e. within each domain)? If not possible then some explanation and concession on how this could have affected the results should be included in the write up.

 

- *In order to address heterogeneity between different trials, we employed random effects models for our network meta-analyses with empirically informative priors for the heterogeneity variance. We did not conduct analyses that were limited to one specific intervention domain, as this approach would have mimicked a pairwise meta-analysis approach. We considered different random-effects model options with or without baseline adjustments or meta-regression based on baseline characteristics and intervention duration. However, we did not find them to affect our treatment estimates nor did they improve the model fit. *

 

It is commonly accepted that interventions' effects vary depending on context. In the realm of this analysis it seems it is not possible to include effect modification analysis and if that is true, the discussion should highlight the impact lack of such analysis may have had on the findings.

 

- *We considered different random-effects model options with or without baseline adjustments or meta-regression based on baseline characteristics and intervention duration. However, we did not find them to affect our treatment estimates nor did they improve the model fit.*

 

I am not entirely clear on the definition of standard of care. In Table 2 this is defined as placebo/no intervention/standard of care/or monotherapy or combination therapy that can be used for indirect comparison. This seems too broad and heterogeneous. Did the authors try narrowing this down further to the placebo/no intervention and rest? How did this very broad categorization affect the findings?

 

- *We thank you for this comment. We have acknowledged this as a limitation in our revised manuscript. We have amended the discussion section to include the following: *

*“Another limitation related to our categorisation of interventions is that we combined interventions into broad categories to assist with interpretation and acknowledge that a different approach to categorisation might have altered the results.”*

# Peer review: Reviewer response for version 1

**DOI:** 10.21956/gatesopenres.14225.r28241

**Published:** 2019-12-13

**Authors:** Tara Cowling, Kelly Larkin-Kaiser, Tayler Scory, Megan Farris

**Affiliations:** 1 Medlior Health Outcomes Research Ltd, Calgary, AB, Canada

In addition to the feedback provided by the other reviewer, we have a few comments for the study author’s consideration.

 The study presented was well conducted, with detailed methods provided in the Supplementary Appendices (SA) demonstrating the rigour of the author’s approach. In addition, the registration of the study protocol on PROSPERO and adherence to the PRISMA statement is a strength of the study.

 The broad research question, with wide-ranging interventions, led to a high volume of evidence being identified. As noted by the authors, the interventions grouped by domain in this analysis would benefit from an analysis based on individual interventions.

 For reproducibility purposes, the manuscript would benefit from clarifying the unexplained discrepancies in the number of trials reported in the SA tables. The PRISMA diagram (Figure 1) states 96 papers were included, representing 79 trials. However, SA Table 4 (list of included studies) lists 91 trials, SA Table 6 (trial characteristics) lists 77 trials, SA Table 7 (patient characteristics) lists 99 trials and SA Table S8 (risk of bias) lists 72 trials. In addition, the asterisk in Figure 3 and 4 should be explained. Finally, in Figure 4 there also appears to be two values of one represented on the x-axis.

 Furthermore, given the large volume of evidence reviewed, the GRADE assessment would be useful to rate the certainty of evidence included in this SLR/NMA. The authors should mention the results of their risk of bias assessment for the included trials to elucidate any biases that might be contributing to the heterogeneity or inform future study designs.

 As detailed in the SA, the authors may also want to state whether or not it was feasible to conduct any meta-regression or subgroup analyses based on effect modifiers in the main manuscript. This would provide the reader with a sense of what might be the source of the heterogeneity and inform future research in this field. Similarly, with mean differences as an outcome, the length of follow-up time may be a source of heterogeneity and no data on the follow-up time is noted in the main manuscript, but it may enhance the interpretation for the reader. Finally, a brief statement on assessing model convergence and inconsistency in the NMA would be informative in the main manuscript for more technical readers. Overall, the statistical methods of the study were robust and thoroughly reported in the SA document.

Are the rationale for, and objectives of, the Systematic Review clearly stated?

Yes

Is the statistical analysis and its interpretation appropriate?

Yes

Are sufficient details of the methods and analysis provided to allow replication by others?

Yes

Are the conclusions drawn adequately supported by the results presented in the review?

Yes

Reviewer Expertise:

Systematic literature review and network meta-analysis.

We confirm that we have read this submission and believe that we have an appropriate level of expertise to confirm that it is of an acceptable scientific standard.

# Response

**Published:** 2020-08-21

**Authors:** Edward Mills

Dear Reviewers:

Thank you for your thorough review of our manuscript. Our responses to your recommendations and comments are marked with bullets below.  

Sincerely,

Edward J. Mills

The broad research question, with wide-ranging interventions, led to a high volume of evidence being identified. As noted by the authors, the interventions grouped by domain in this analysis would benefit from an analysis based on individual interventions.

 

- *Thank you for your comment. The Supplementary Crosstable – v1.0 that we provided captures interactions between individual interventions. The Bayesian network analysis tool that was used to create the cross table can also be used to report analysis based on individual intervention. Given the nature and objective of our paper, we provided a comprehensive framework where comparative effectiveness of multiple interventions under multiple treatment domains can be understood. *

 

For reproducibility purposes, the manuscript would benefit from clarifying the unexplained discrepancies in the number of trials reported in the SA tables. The PRISMA diagram (Figure 1) states 96 papers were included, representing 79 trials. However, SA Table 4 (list of included studies) lists 91 trials, SA Table 6 (trial characteristics) lists 77 trials, SA Table 7 (patient characteristics) lists 99 trials and SA Table S8 (risk of bias) lists 72 trials. In addition, the asterisk in Figure 3 and 4 should be explained. Finally, in Figure 4 there also appears to be two values of one represented on the x-axis.

 

- *Thank you for pointing out the discrepancy. The PRISMA chart shows that we have 96 papers as final includes which consist of both primary and secondary studies. Among the 96 papers, there are 79 unique trials, that is, they exclude secondary studies with the same trial ID. SA Table 4 has now been updated to reflect 96 studies. Please note that the paper Zambrana 2019 is one single primary study, reporting on two separate trials with unique trial registries. As a result, it has been captured as two trials on SA Table 4. Menasria 2018, which was initially part of the final includes, was not included in our analysis since the intervention arms did not consist the usual food/dietary supplements and thus represented as an outlier. Hence, the number of unique trials that were part of the analysis was 78. The updated SA table can be found here:* *https://doi.org/10.17605/OSF.IO/DTZK7* *. *
- *SA Table 6 includes characteristics from 77 trials. The two trials from Zambrana 2019 have been combined into one here, both clinical trials from this study share similar study characteristics, which is why the number is 77. *
- *SA Table 7 has been updated to reflect 78 trials.*
- *SA Table 8 has been updated to reflect 77 studies. You can find the updated table here* *https://doi.org/10.17605/OSF.IO/DTZK7* *.*
- *Explanations for Figures 3 & 4 have been updated. The horizontal axis for Figure 4 has been edited. *

 

Furthermore, given the large volume of evidence reviewed, the GRADE assessment would be useful to rate the certainty of evidence included in this SLR/NMA. The authors should mention the results of their risk of bias assessment for the included trials to elucidate any biases that might be contributing to the heterogeneity or inform future study designs.

 

- *Thank you for your suggestion. We have updated the bias section of the manuscript to include the following:*

*“Bias was evaluated using Cochrane risk-of-bias assessment tool* *in the areas of selection, performance, detection, attrition, reporting, and other sources of bias. Over 60% of the studies exhibited low bias in terms of attrition, selection, and reporting bias. Sources of detection and performance bias were unclear in about 25-30% of the studies.”*

As detailed in the SA, the authors may also want to state whether or not it was feasible to conduct any meta-regression or subgroup analyses based on effect modifiers in the main manuscript. This would provide the reader with a sense of what might be the source of the heterogeneity and inform future research in this field. Similarly, with mean differences as an outcome, the length of follow-up time may be a source of heterogeneity and no data on the follow-up time is noted in the main manuscript, but it may enhance the interpretation for the reader. Finally, a brief statement on assessing model convergence and inconsistency in the NMA would be informative in the main manuscript for more technical readers. Overall, the statistical methods of the study were robust and thoroughly reported in the SA document.

 

- *We thank you for this comment. Given the limited evidence base, meta-regression analyses of different potential effect modifiers were not possible. We have checked the model fit using leverage plots and DIC and consistency assumption using consistency plots of indirect and direct evidence. These results can be found in the Supplementary File (Supplementary Figures 5-8). *

# Peer review: Reviewer response for version 1

**DOI:** 10.21956/gatesopenres.14225.r28242

**Published:** 2019-11-26

**Authors:** Judd L. Walson

**Affiliations:** 1 Department of Global Health, University of Washington, Seattle, WA, USA

This is a very interesting study describing the results of a network meta-analysis of studies of interventions to improve HAZ (and therefore reduce stunting in LMIC settings). There are a number of issues that should be addressed in the manuscript:

1. I find it difficult to reconcile the differences observed in the analyses between changes in HAZ and changes in stunting. I would have expected that the analysis incorporating a continuous variable (HAZ) would have had more power to detect differences than the analysis of stunting as a dichotomous variable? If this is so, what is a reasonable explanation for the finding that interventions would have an impact on stunting but not on HAZ (given that stunting is a function of LAZ)?
2. It would be useful to comment on the clinical relevance of these findings. What would be the expected population level impact on developmental outcomes or survival based on these effect sizes?
3. The authors adhered to the PRISMA guidelines, which is excellent. However, there are few unclear issues with the methods. It appears that the authors searched reviews but then abstracted individual trials? Did the search also focus on individual trials or where these only identified through reviews? If individual trials were included, then these should be listed.
4. Is it possible to include the I ^2^ with the forest plots?
5. I am concerned by the blanket use of am ICC of 0.05. Would it not be preferable to estimate the ICC for each individual study based on similar studies in that environment or with a similar design effect? Perhaps categorizing into 5 different ICCs that could be applied to each? At the very least, it would be useful to conduct a sensitivity analysis where the ICC is varied.
6. The authors note that flour appeared to have an effect on stunting. However, the 95% CI includes 1.0. Should this be considered a significant finding?
7. Some of the grammar in the manuscript could be improved, particularly in the abstract. Please edit for grammar.

Are the rationale for, and objectives of, the Systematic Review clearly stated?

Yes

Is the statistical analysis and its interpretation appropriate?

Partly

Are sufficient details of the methods and analysis provided to allow replication by others?

Yes

Are the conclusions drawn adequately supported by the results presented in the review?

Partly

Reviewer Expertise:

infectious disease, nutrition, trials in LMIC settings

I confirm that I have read this submission and believe that I have an appropriate level of expertise to confirm that it is of an acceptable scientific standard, however I have significant reservations, as outlined above.

# Response

**Published:** 2020-08-21

**Authors:** Edward Mills

Dear Dr. Walson:

Thank you for your thorough review of our manuscript. Our responses to your recommendations and comments are marked with bullets below.  

Sincerely,

Edward J. Mills

I find it difficult to reconcile the differences observed in the analyses between changes in HAZ and changes in stunting. I would have expected that the analysis incorporating a continuous variable (HAZ) would have had more power to detect differences than the analysis of stunting as a dichotomous variable? If this is so, what is a reasonable explanation for the finding that interventions would have an impact on stunting but not on HAZ (given that stunting is a function of LAZ)?

 

- *Thank you for your comment. The observed differences between the impact on HAZ and stunting can be attributed to randomness in the data as well as substantial heterogeneity observed in the duration of the interventions and the timing of outcome assessments.* *We have updated the discussion section to include this as a limitation. *

It would be useful to comment on the clinical relevance of these findings. What would be the expected population level impact on developmental outcomes or survival based on these effect sizes?

 

- *We have pointed out in the discussion section that the existing evidence base is limited and does not provide a robust answer in regards to how food and other interventions, such as deworming, maternal, and WASH, impact linear growth outcomes in children in LMICs. As such, there is no strong evidence to report on population level impact on developmental outcomes. *

The authors adhered to the PRISMA guidelines, which is excellent. However, there are few unclear issues with the methods. It appears that the authors searched reviews but then abstracted individual trials? Did the search also focus on individual trials or where these only identified through reviews? If individual trials were included, then these should be listed.

 

- *Our search started with a review of systematic reviews before finalizing the study scope in terms of PICOS and the search strategies. For this, we performed a hand-search of the bibliography of Bhutta 2013 to identify relevant systematic reviews and trials, and searches were done on PubMed and the Cochrane Database of Systematic Reviews to identify additional reviews that were published after 2013. The list of published reviews relevant to this study is provided in Table 1. *
- *For the individual randomized clinical trials for our meta-analyses, a comprehensive search of literature was conducted from the Cochrane Central Register of Controlled Trials, Embase, and MEDLINE database to identify relevant trials and any additional relevant reviews that were missed in the prior step. The database search was complemented by bibliographical review of the published literature reviews to ensure that we did not miss any important clinical trials in our evidence base. The list of included and excluded trials are available in the Extended data (Supplementary Table 4 for included and Table 5 for the list of excluded studies). Here is the URL for your reference:* *https://doi.org/10.17605/OSF.IO/DTZK7* *. *

 Is it possible to include the I ^2^ with the forest plots?

 

- *In order to address heterogeneity between different trials, we employed random effects models for our network meta-analyses. We used empirically informative priors for the heterogeneity variance for binary outcomes and non-informative priors for continuous outcomes. Since the use of I ^2 ^as a measure of heterogeneity in Bayesian network meta-analysis is uncommon, we have decided to exclude it for the purpose of this study.*

I am concerned by the blanket use of am ICC of 0.05. Would it not be preferable to estimate the ICC for each individual study based on similar studies in that environment or with a similar design effect? Perhaps categorizing into 5 different ICCs that could be applied to each? At the very least, it would be useful to conduct a sensitivity analysis where the ICC is varied.

 

- *Thank you for this comment. Within the cluster trials included in our network meta-analysis, an average value of ICC of 0.0505 was reported* *, so we assumed a conservative value of 0.05 to adjust for the clustering effects of the cluster trials in our analyses. The ICC was used to inflate variance accordingly for the continuous outcome and to down-adjust the sample sizes and the number of cases for the dichotomous outcome, as recommended by Uhlmann 2016 (* *https://pubmed.ncbi.nlm.nih.gov/27390267/* *). It is expected that in a network meta-analysis with cluster randomized trials, a varying level of ICC would not impact/change results. *

The authors note that flour appeared to have an effect on stunting. However, the 95% CI includes 1.0. Should this be considered a significant finding?

 

- *The reason why we reported this is because flour showed some reduction in stunting compared to standard of care in contrast to other interventions whose CI completely crossed the null effect of 1. *

Some of the grammar in the manuscript could be improved, particularly in the abstract. Please edit for grammar.

 

- *This is now corrected. *
